# Direct mineralogical imaging of economic ore and rock samples with multi-modal nonlinear optical microscopy

**DOI:** 10.1038/s41598-018-34779-9

**Published:** 2018-11-16

**Authors:** Mung-Chung Kao, Adrian F. Pegoraro, David M. Kingston, Albert Stolow, Wen-Chuan Kuo, Patrick H. J. Mercier, Ankur Gogoi, Fu-Jen Kao, Andrew Ridsdale

**Affiliations:** 1 Institute of Biophotonics, National Yang Ming University, 155, Sec. 2, Li-Nong St., Taipei, 112 Taiwan; 2 Department of Physics, University of Ottawa, 25 Templeton Ave., Ottawa, ON K1N 6N5 Canada; 3 National Research Council Canada, 100 Sussex Drive, Ottawa, ON K1A 0R6 Canada; 4 Department of Chemistry, University of Ottawa, 10 Marie Curie, Ottawa, ON K1N 6N5 Canada; 5 Department of Physics, Jagannath Barooah College, Jorhat, 785001 Assam India

## Abstract

Multi-modal nonlinear optical (NLO) microscopy, including stimulated Raman scattering (SRS) and second harmonic generation (SHG), was used to directly image mineralogical features of economic ore and rock samples. In SRS/SHG imaging, ore samples generally require minimal preparation and may be rapidly imaged, even in their wet state. 3D structural details, at submicron resolution, are revealed tens of microns deep within samples. Standard mineral imaging based on scanning electron microscopy (SEM), with elemental analysis via energy dispersive X-Ray spectroscopy, was used to independently validate the mineral composition of the samples. Spatially-resolved SRS from dominant Raman-resonant bands precisely maps the locations of specific minerals contained within the samples. SHG imaging reveals locally non-centrosymmetric structures, such as quartz grains. Competing absorption and nonlinear scattering processes, however, can reduce contrast in SRS imaging. Importantly, the correlation between standard electron microscopy and multi-modal NLO optical microscopy shows that the latter offers rapid image contrast based on the mineral content of the sample.

## Introduction

The need for high quality, real-time information in mineral resource characterization is growing due to both declining ore grades and demands for improved energy efficiency. Low grade ore and source rocks have highly complex mineralogy and textures which impact mineral processing and extractive metallurgy. This challenge prompted the rapid development and commercial deployment of microscopic imaging technologies for characterizing mineral ores and source rocks, including high-resolution X-ray computed tomography (microCT), neutron computed tomography, scanning electron microscopy (SEM) based automated mineralogy, etc., which allow rapid characterization and identification of key mineral phases in ore/rock samples^1–4^. Despite the analytical capability of these advanced methods for efficient mineral characterization, significant uncertainties persist for each. For example, both mineral liberation analysis (MLA) and quantitative evaluation of minerals by scanning electron microscopy (QEMSCAN) are scanning electron microscopy (SEM) based techniques and, as with other electron-beam techniques, cannot distinguish between minerals having very similar elemental composition (e.g., hematite and magnetite) or mineral polymorphs (e.g., the titania polymorphs rutile‒anatase‒brookite)^5–7^. Nevertheless, judicious use of these techniques in conjunction with complementary techniques, e.g., optical microscopy^4^, cathodoluminescence (CL) microscopy and spectroscopy^8^, X-Ray fluorescence^9^, infrared thermography^10^, *etc*., may overcome these limitations and significantly enhance the quality and reliability of the results. This is illustrated by digital optical microscopy (DOM) which can distinguish ores consisting of minerals of similar elemental composition, e.g. hematite and magnetite^11^. Indeed, DOM was recently successfully integrated with MLA and QEMSCAN automated mineralogy techniques^12^. Such microscopy approaches are often referred to as correlative microscopy (sometimes also referred as co-site, collaborative or co-localization microscopy^13–15^) and have been utilized in a range of studies in both biology^16–18^ and hydrology^19^.

Most efforts in correlative microscopy are based on standard linear optical methods. Correlative microscopy has yet to take advantage of important developments in nonlinear optical (NLO) microscopy which offer valuable new contrast mechanisms for many systems, with some inherent advantages over traditional linear optical techniques. Certain NLO processes can be insensitive to the specific colour of input light, allowing longer (near IR) wavelengths to be used. This reduces both absorption and scattering, thereby increasing penetration depths, even in wet samples. Furthermore, since nonlinear signals are generated only at the focus, these methods are inherently confocal, with high 3D spatial resolution. Different NLO signals can be complementary and measured simultaneously (along with other NLO processes) with a single multimodal microscope^20–22^ with different NLO signals revealing distinct structural-chemical details. For example, second harmonic generation (SHG) can only occur in locally non-centrosymmetric systems, revealing details of atomic structure and orientation^23,24^. While most applications of SHG microscopy are in biology and medicine, there are several examples of SHG imaging in studies of the structure of metallic and semiconductor surfaces, and in monitoring processes at mineral oxide/liquid interfaces^20,25–27^. An important NLO imaging technique is coherent Raman scattering (CRS) microscopy, which includes both coherent anti-Stokes Raman scattering (CARS) and stimulated Raman scattering (SRS) microscopy. CRS techniques are NLO versions of spontaneous Raman scattering, a technique which has emerged as a powerful tool for non-destructive extraction of vibrational and chemical-specific information from a wide range of mineral specimens, ranging from lump ore to fine powders and solid-liquid slurries^28–33^. Unfortunately, spontaneous Raman scattering signals are typically very weak and can be overwhelmed by background fluorescence signals, limiting its use in correlative microscopy analysis. In contrast, CRS imaging is typically immune to fluorescence and can be much faster than spontaneous Raman imaging, thus enabling a wide range of real-time imaging applications^34,35^. The advantages of CRS - particularly SRS - over traditional Raman microscopy have led to its dramatic growth as a tool for label-free molecule-specific imaging in biomedicine^36,37^. Recently, in a proof-of-concept study, SRS was applied to 3D chemical-specific imaging of minerals^20^. To be relevant, however, to mineralogy and the Earth Sciences in general, SRS imaging must be demonstrated and proven on samples of economic interest.

Here we extend and, importantly, validate our approach by directly comparing SEM with SRS/SHG microscopy in ores of economic interest, thus demonstrating that multi-modal NLO microscopy offers a potentially powerful new tool for advanced rapid mineral processing analytics. We apply a correlative microscopy methodology which combines images obtained from both SEM and multimodal NLO microscopy to map mineral features of various economically-relevant ore and other types of rock samples. Both thin sections and ground particulate samples were investigated. SEM coupled with energy-dispersive X-ray spectroscopy (EDS) allows for the detection of specific atomic elements within the minerals and substrate. In contrast, SRS imaging allows spatially-resolved mapping of specific minerals based on their dominant Raman bands. SHG microscopy allows the mapping of locally non-centrosymmetric mineral phases (e.g. quartz particles) within the samples. Important in process mineralogy applications, samples for SRS/SHG imaging (unlike SEM-EDS methods) generally require minimal preparation and may be rapidly imaged even in their wet state. This correlative microscopy approach provides more information about the sample than can be achieved by any single technique alone and, importantly, validates the SRS/SHG imaging approach. In Table 1, we compare important aspects of SRS, SHG and SEM-EDS systems.

**Table 1:** Important aspects of SRS, SHG and SEM-EDS systems.

Technique	Method	Strengths	Limitations
Stimulated Raman scattering (SRS) microscopy	This NLO microscopy uses two ultrashort pulse lasers, tuned to probe a Raman shift of interest.	Non-destructive, non-contact, label free, rapid, micron scale resolution, intrinsic vibrational contrast, 3D optical sectioning capacity, deeper penetration if near IR wavelengths are used, no need for sample preparation, applicable to solid, wet, liquid as well as gaseous samples	Not sensitive to metals and alloys, difficult to discriminate between materials with overlapping spectral features, possibility of fluorescence interference, absorption and other strong interaction with samples such as nanoscale objects which can produce interference
Second harmonic generation (SHG) microscopy	This NLO microscopy technique using a single ultrashort laser and is sensitive only to regions containing non-centrosymmetric species.	Non-destructive, label free, micron scale resolution, high sensitivity and selectivity of SHG in non-centrosymmetric structures, 3D optical sectioning capacity, deeper penetration if near IR wavelengths are used, no need for sample preparation	Limited applicability since SHG signals are generated only from non-centrosymmetric structures. Additionally, lacking inversion symmetry does not guarantee the presence of SHG signals.
Scanning electron microscopy - energy dispersive X-ray spectroscopy (SEM-EDS)	In this method a focused beam of high-energy electrons is used to generate a variety of signals including secondary electrons, backscattered electrons, characteristic X-rays, cathodoluminescence etc., at the surface of the specimens.	Offering the highest possible magnification and resolution, data acquisition is rapid (typically few minutes/image for SEI, BSE, spot EDS analyses), full elemental spectrum can be obtained within a few seconds by using spot EDS analysis	Samples must be solid, with relatively time-consuming and costly sample preparation process. Restricted to surfaces only. Wet samples are not possible. The EDS energy peaks of different elements may overlap

SEI: secondary electron image; BSE: back-scattered electrons; EDS: energy-dispersive X-ray spectroscopy.

## Results and Discussion

### Multimodal mapping of mineral features with NLO microscopy

**Figure 1:**
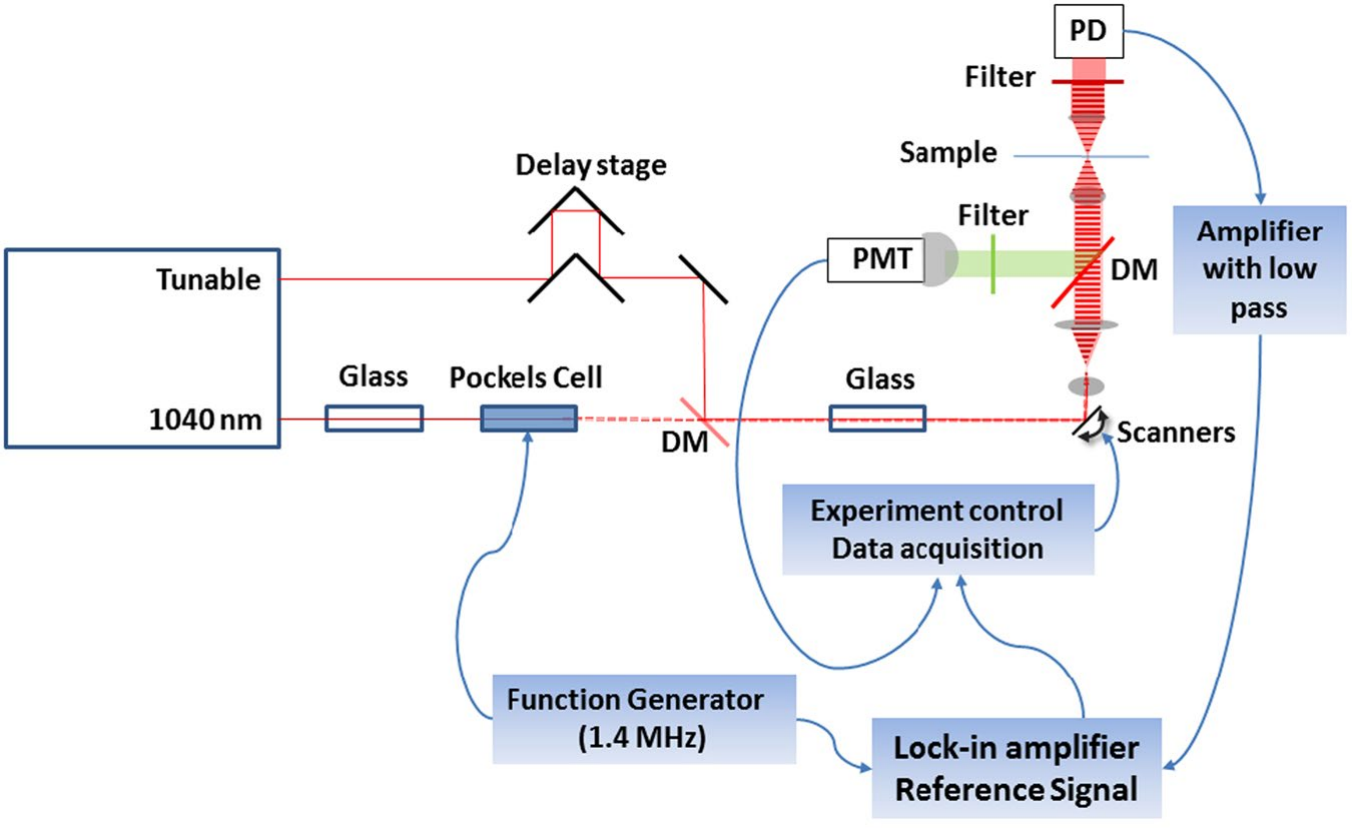
Layout of the multimodal NLO microscopy apparatus. Spectra-Physics Insight Laser provides two synchronized pulsed laser beams, one at 1040 nm and another tunable from 690–1300 nm. The tunable beam travels through a delay stage which allows the high resolution adjustment of the inter-pulse timing and hence changing the Raman mode probed. The 1040 beam is modulated at 1.4 MHz by a Pockel’s cell which is driven by a function generator that also provides the reference for the lock-in amplifier. Both pulses traverse pieces of high refractive index glass which causes the femtosecond pulses to be stretched to an overlapped envelope of around 2 ps which improves spectral resolution and enables rapid spectral scanning. The two beams are combined on a dichroic mirror (DM) before entering the laser scanning microscope. For SRS measurements, the modulated 1040 beam is blocked by using filters after passing through the sample, whereas the tunable beam is detected by a photodiode (PD). This signal is amplified and low-pass filtered by a transimpedance amplifier before going to the signal input of the lock-in amplifier. For SHG detection, another dichroic mirror is used in the backward direction to direct the back-reflected SHG signals through a bandpass filter and detected by a photomultiplier tube (PMT).

A schematic of the experimental apparatus is shown in Fig. 1. SRS relies on coupling (modulation transfer) between two ultrashort pulse lasers whose frequency difference is tunable. One beam is modulated and the measured signal is the transfer of modulation to the other laser beam. When their frequency difference matches a Raman mode in the sample, energy transfer takes place between the beams, resulting in modulation transfer. The Raman spectrum is scanned by: (i) tuning the central frequency of one beam and/or (ii) changing the temporal overlap between the beams via the chirped-pulse spectral focusing method^38–40^. The signal generated from SHG arises from a single beam and is detected by using a second dichroic mirror located in the backward direction, selectively directing the visible SHG emission from the sample toward a photomultiplier tube (PMT). Further technical details regarding the laser scanning microscopy and the ultrafast laser used are presented in the section “Laser scanning microscopy” in Materials and Methods.

### Three dimensional mapping of ground minerals

**Figure 2:**
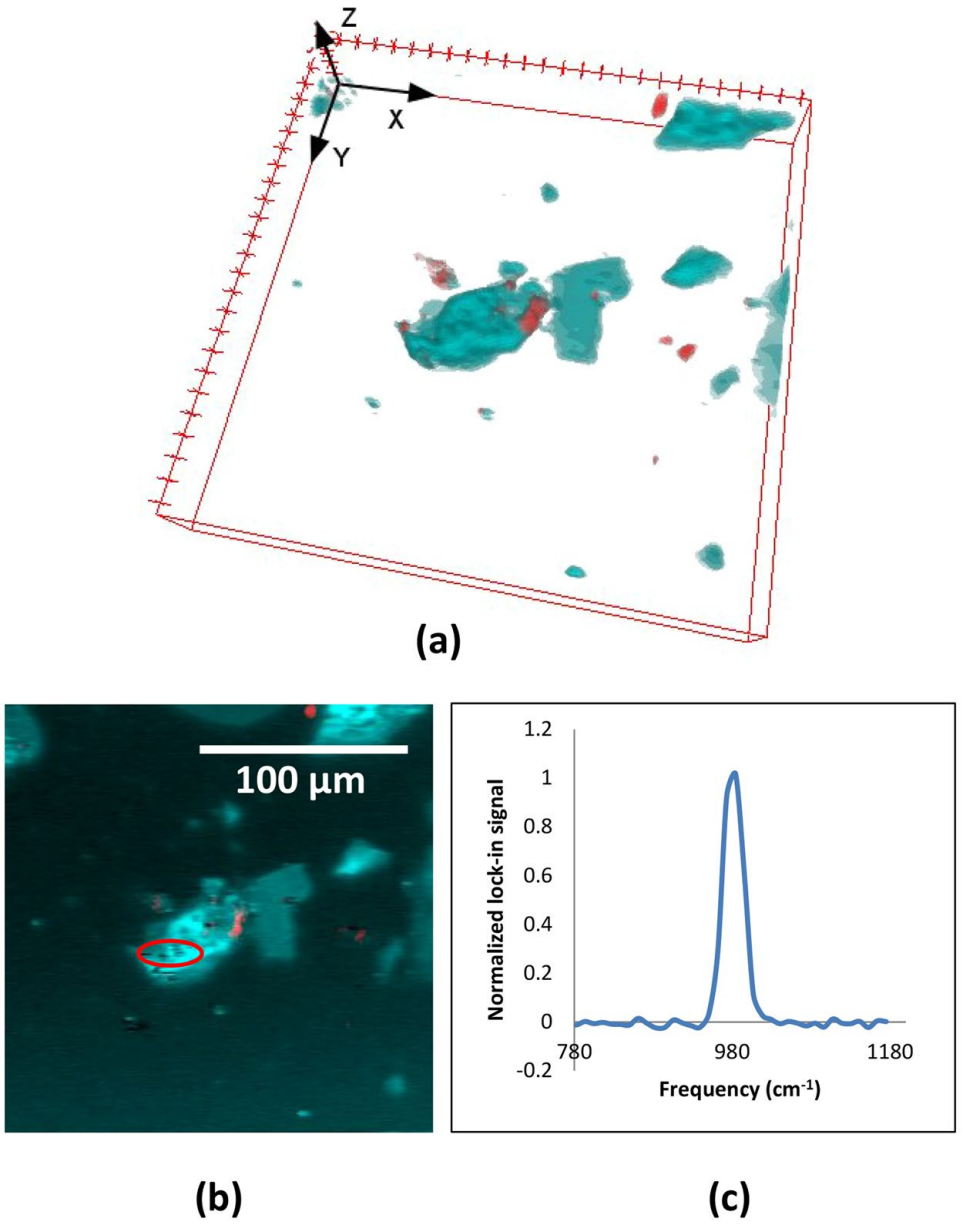
Natural barite (BaSO_4_) ore sample, ground in a mortar and pestle, was mostly transparent grains with a fair number of pink/red components. Analysis by powder X-Ray diffraction indicates the presence of other minerals such as quartz and cerusitte (PbCO_3_). Grains were placed on a coverslip and a drop of cyanacrylate glue was added on top, then placed on a slide. The cyanoacrylate was allowed to cure for about 1 hour before imaging. Laser conditions were 40 mW at 943 nm and 20 mW at 1040 (modulated). (**a**) The strong backward propagating signal (shown in red) spatially overlaps, consistently, with the red colored grains in the sample, whereas the forward-collected SRS signal (shown in cyan) is tuned to the sulfate Raman resonance of BaSO_4_ at 985 cm^−1^. Volume is 205 × 205 × 40 microns into a 256 × 256 × 40 voxel data set. Focal slices were collected at 1 micron intervals. The total data collection time was about 90 s. (**b**) 2D image of the Raman spectral scan of the region of interest (red oval), confirms the BaSO_4_ Raman resonance at 985 cm^−1^, shown in (**c**).

Mapping of the mineral content in ground ores provides important information in the planning of ore processing. Some of the most valuable parameters derived from microscopy relate to the surface exposure of mineral types on grains (mineral liberation). A potential advantage of a NLO laser scanning techniques over SEM-based approaches is that the three spatial dimensions can be mapped in reasonably thin and transparent samples, providing better surface exposure metrics which complement those obtained with relative difficulty and sample-dependence using 2D imaging^41–44^. To demonstrate the possibilities of using NLO microscopy for mapping of 3D mineral grains, we ground naturally occurring barite (BaSO_4_) before embedding in a cyanoacrylate mixture for imaging. We imaged a 3D volume and recorded all backward propagating signals (red) and a forward propagating SRS signal (cyan) tuned to the sulfate peak of barite around 985 cm^−1^ (Fig. 2, top). The backward propagating signal spatially correlates with red/pink colored grains that can be seen using transmitted light. Rietveld refinement of the X-ray diffraction pattern generated from the powdered sample indicates that it contains about 12% quartz and 17% cerussite (PbCO_3_), as well as 69% barite. To confirm that SRS contrast is derived from the sulfate peak, we performed a spectral scan in a single 2D plane of the volume and recovered the expected Raman response (Fig. 2, bottom). We can tentatively assign the backward signal as SHG from quartz particles contained in the sample. The intensity of the SHG response is spatially inhomogeneous within natural quartz grains; however, the SHG signal is very strong and if some voxel elements are saturated in the image processing, we find that the reconstructed grain shape matches the grain size when compared to transmitted light images. Note that the full quartz grain could be definitively mapped by tuning the SRS system to the 466 cm^−1^ peak of quartz, but this would double the required collection time.

### Rapid mapping of major mineral types in commercially significant rare earth element ores

**Figure 3:**
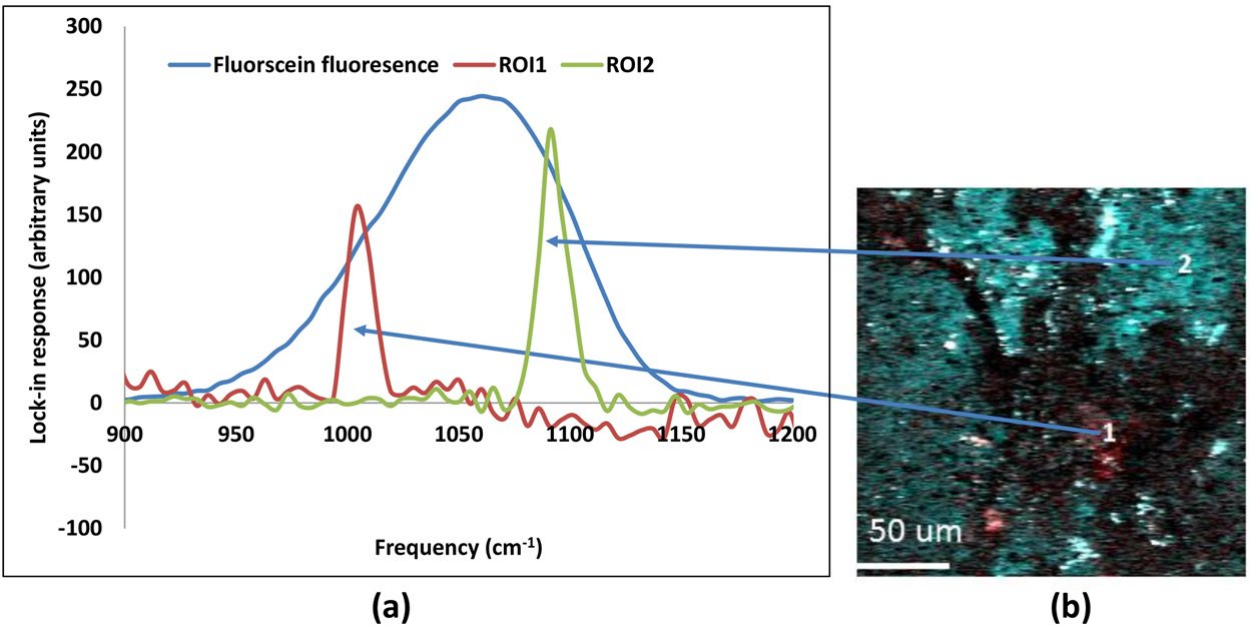
(**a**) Raman mapping of SRS responses from the regions of interest (ROI) within a sample of carbonatite rare earth ore shown in image (**b**). For reference, the non-degenerate two-photon excited fluorescence from fluorescein solution (collected separately) maps the cross correlation of the pump and Stokes pulses at the sample and provides a normalizing factor for the Raman peak response (note this signal is not collected from the lock-in and is on a different scale). The sulfate Raman response (~1005 cm^−1^) is shown in red. The response of calcite (~1090 cm^−1^) is also indicated. The original hyperspectral data set is 256 × 256 image pixels with 94 spectral data points. Images across the peak regions are averaged and used to make the red (around 1005 cm^−1^) and green-blue (cyan-1090 cm^−1^) color merged image. Note the dynamic range of this image has been narrowed so as to show the spatial extent of the SRS signals. Very strong signals exhibit as bright points (grey/white) as well as black areas of the image. These signals are generally higher in amplitude than SRS and show different responses to the delay stage position.

Given appropriate prior information on the mineral types likely present in the ore, a strategy to rapidly map mineralogical regions within the sample can be developed. As an illustrative example, shown in Fig. 3, we imaged a thin section of carbonatite rare earth ore containing both sulfate and carbonate rich components. Using spectral scanning SRS, different mineralogical regions can be identified based on their Raman spectra, as is shown in Fig. 3(a). The sulfate peak at 1005 cm^−1^ and the carbonate peak at 1090 cm^−1^ are clearly identifiable in different regions of interest (ROIs). Using the natural Raman resonance for image contrast, we can visualize the mineralogical content of the sample. Here, using red to identify sulfate and cyan to identify carbonate, we can see the spatial heterogeneity within the sample, shown in Fig. 3(b).

Interestingly, not all of the signals corresponding to the red and cyan channels in Fig. 3(a) are Raman resonant. It is well known that linear and nonlinear absorption, as well as nonlinear scattering, can appear as a background signal in SRS microscopy^45,46^. In certain spatial regions within these samples, there are very strong responses due to transient absorption or photo-thermal responses; in many ore samples, these nonresonant signals arise from linear absorption. Importantly using more Raman spectral data points in combination with SHG, we were able to distinguish various mineral responses without a full spectral scan. Such sparse spectral sampling will be necessary to realize the potential for high-throughput screening applications^47^. Classification of hyperspectral data from sparse data sets is an area of current active research but is not addressed here. The SRS signal is proportional to the time overlap of the two beams, shown here by the fluorescence response of fluorescein. The full width half maximum (FWHM) of the overlap, in frequency units, is around 105 cm^−1^. This two-photon fluorescence response provides the normalization factor which allows comparison of the Raman signals at various frequencies. In crystalline materials, this response is also dependent on the sample alignment with respect to the polarization of the input laser fields and is generally not controlled in the preparation of ore and rock samples. The non-degenerate two-photon excitation response tends to be somewhat asymmetrical (in contrast to the sum frequency generation in KDP crystals), likely due to the spectral dependence of the fluorescence.

### Correlative/co-localization microscopy

**Figure 4:**
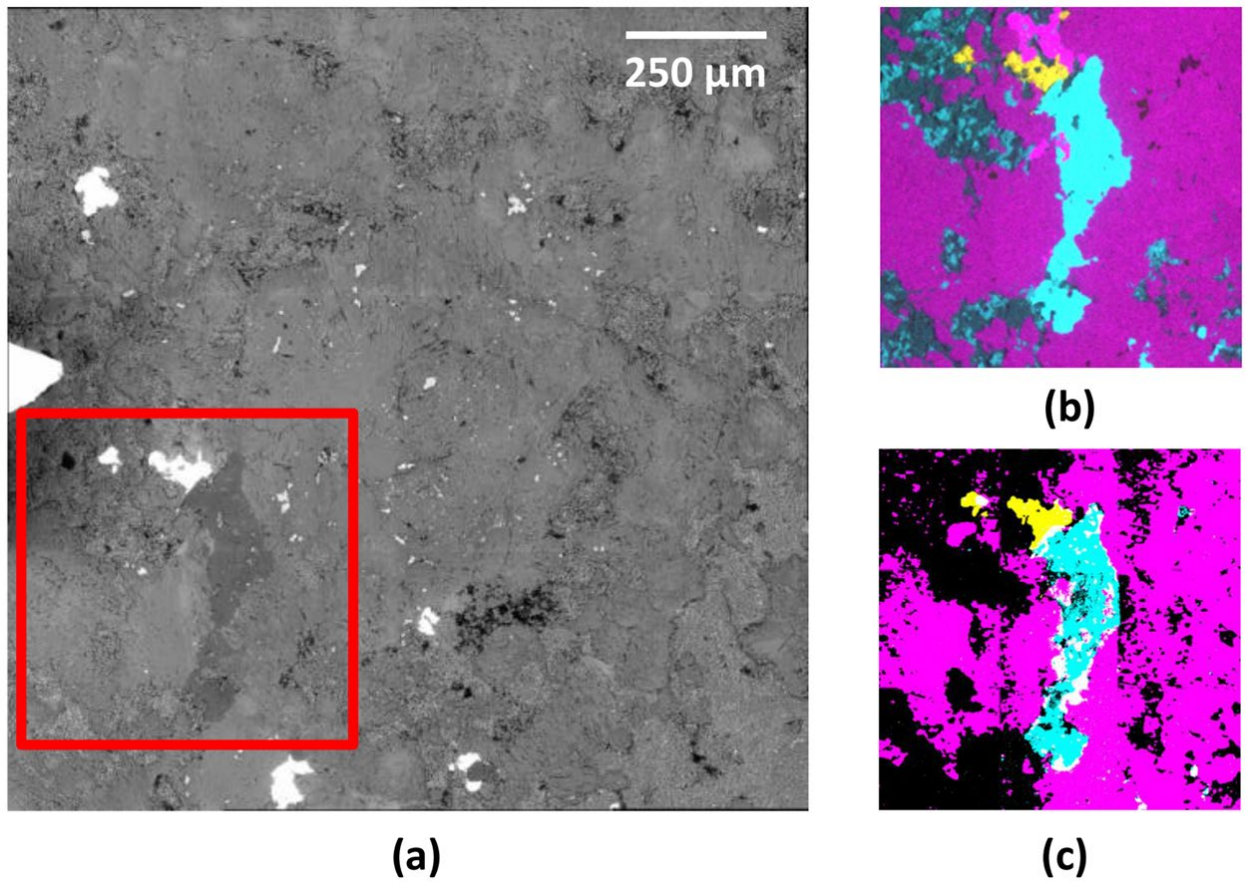
Correlative SEM/EDS and NLO (SRS and SHG) imaging. (**a**) A large area back scattered electron image of a thin section from a rare earth element bearing carbonatite ore, with the region of interest to be compared with SRS/SHG imaging outlined in red. (**b**) An overlay of SEM-EDS elemental analysis. Si is colored in cyan, Ca in magenta, and S in yellow. Comparing with other elemental maps allowed us to identify the cyan region as quartz, the magenta region as ankerite containing CO_3_, and the yellow region as barite containing SO_4_. (**c**) An SRS spectral scan with simultaneous SHG imaging, identifying several different regions. This composite image was formed by stitching together 9 fields of views before binarizing the images to identify different regions. The SHG (i.e. quartz) represented by cyan, a carbonate Raman mode centered around 1090 cm^−1^ (CO_3_) colored as magenta, and a sulfate Raman mode at 985 cm^−1^ (SO_4_) represented by yellow. It can be seen that the global spatial correlation between (**b**,**c**) is very good, confirming that SRS/SHG imaging can be used for mineralogical analysis of economic ores.

While multimodal NLO microscopy can clearly offer insights into the mineralogical structure of the sample, these must be validated against standard SEM-EDS techniques. To demonstrate this, we used a sample of rare-earth element carbonatite ore for correlative microscopy and co-location analysis. Elemental mapping analysis by SEM-EDS revealed ankerite (Ca(Fe,Mg,Mn)(CO_3_)_2_), barite (BaSO_4_) and quartz as the main components. We restricted our area of analysis to the region around a quartz grain to better enable comparisons between techniques, as quartz should offer a strong, distinct SHG response which can be used to cross-validate these techniques. The backscattered electron image, for a restricted field of view, is shown in Fig. 4(a). Based on elemental analysis, we expected both carbonate (1090 cm^−1^) and sulfate (1005 cm^−1^) SRS Raman resonances, as well as SHG signals from the quartz phase. Note that, as in Fig. 3, the SRS image was processed to reveal the spatial variation of the Raman response. In Fig. 4, however, strong point-like background signals (non-Raman resonant) were subtracted by averaging 10 scans with the laser set spectrally just outside the Raman resonance: the resulting images were binarized to ease comparison. NLO imaging has a restricted viewing area for collecting a single micrograph at high resolution, but several fields of view can be stitched together to form a composite image, thus allowing direct comparisons between SEM and NLO imaging over a larger sample area. SHG is expected only from the quartz phase and this should match the Si elemental map. Therefore, we used this correlation to spatially align the SEM and NLO images (there was no automatic alignment between SEM and SRS imaging in our current set-up). Using a blind image transformation algorithm, the SHG image was translated, rotated, and scaled to achieve maximum spatial correlation with Si elemental map. This transformation was then applied to the SRS images. The spatial correlation between the binary images from elemental analysis, Fig. 4(b) and those from the SRS imaging, Fig. 4(c), was then determined. We found that the correlation between the Ca elemental map and the carbonate SRS image was 80% over the field of view (pixel by pixel analysis), whereas the S/SO_4_ agreement was 92%. To shed light on the residual discrepancy between SEM and SRS images, we overlaid Fig. 4(b) (i.e. Si, Ca, and S images) and 4(c) (i.e. SHG, CO_3_, and SO_4_ images). This revealed that the largest deviations are along the edges of the composite image. This likely caused by a slight tilt of the 2D image scan plane for SRS, relative to the sample surface. In NLO microscopy, the signal is strongly localized within the microscope focus and even small (micron scale) deviations of the sample plane relative to the 2D scan plane can lead to a systematic variation in signal intensity across the full composite image. Another general source of discrepancy between Fig. 4(b,c) could arise from the fact that SEM is uniquely surface (2D) sensitive whereas SRS signals obtain from a depth (3D) of several microns. These small, residual discrepancies require further quantification and will be the subject of our future studies. Nevertheless, it is clear that the overall agreement in the general features is quite robust.

A key advantage of SRS over spontaneous Raman is its relative insensitivity to sample fluorescence, an aspect which may be of high value in certain applications. Nevertheless, it is important to consider image acquisition speeds of different modalities because the high throughput screening of many particles would be of great value in the planning of mine operations. The SEM-EDS spectra were collected here were at around 2 ms per image (depending on the electron beam current and other factors determining count rate above background): this is faster than standard (spontaneous) Raman by a factor of 100 or so. Although SRS signals can be much stronger than spontaneous Raman, direct comparisons of image acquisition speed, with meaningful figures of merit, are still difficult to obtain. In the comparison of spontaneous Raman to SRS, the time required for collecting broad spectra at similar signal-to-noise ratios is of a similar order^48–50^. This is typically because of sample-damage-limitations in SRS due to laser absorption. Moreover, if collection of the entire Raman spectrum is required, the spectral focusing implementation used here suffers in comparison to spontaneous Raman since the lowest Raman frequencies (<300 cm^−1^) are not easily accessible in SRS. On the other hand, if monitoring of only a few selected individual Raman bands is sufficient for analysis, the SRS approach potentially enables much faster imaging in a multimodal/sparse sampling regime^38,51^. In this case, only a few Raman modes (rather than the entire spectrum) are used in imaging, greatly increasing the imaging speed. The same argument applies to the combination of SRS with SHG imaging. For example, the image of mineral grains shown in Fig. 2 was sampled at around 35 microseconds per voxel, with good signal to noise ratios and without sample damage, showing the mapping of two mineral species in 3D. This represents a near-ideal case where we simultaneously collected two diagnostic data channels.

## Conclusions

We have investigated the use of multi-modal NLO microscopy, a technique largely developed for biomedical imaging, in its applications to the process mineralogy of economic ores. Through the combination of SEM-EDS with NLO (SRS/SHG) imaging, we have demonstrated the value of correlative microscopy for geo-photonic/geo-metallurgy applications by presenting elemental, chemical and structural images of the minerals found in the selected ore and rock samples. The information contained in the correlation exceeds that of either technique alone. A mere co-localization of the elements may not indicate the presence of a specific mineral phase. For example, not all carbon (C) in the elemental map is due to carbonate (CO_3_). This illustrates why a correlative modality which combines SEM-EDS with NLO (SRS/SHG) imaging could be of benefit in mineralogy applications. The comparison presented in Fig. 4 demonstrates that SRS/SHG microscopy can be used to image mineral phases in ores of economic interest.

A key advantage of the NLO modalities demonstrated here is that powdered (even wet) material can be imaged without further preparation. This is especially the case when 3D, rather than 2D surface, imaging is most valuable. Furthermore, without the need for extensive sample preparation, SRS imaging is likely to work in a rougher field environment than does SEM-EDS and could therefore provide much faster turnaround. Our data demonstrated quite rapid mapping of areas within thin sections. Finally, SHG and, in some cases, fluorescence signals may provide additional contrast that can aid in the unambiguous assignment of mineral types.

It is important to note, however, that background signals and laser-induced sample damage issues need to be considered in NLO imaging. In addition to SRS/SHG signals, we observed many strong responses from most ore samples tested which appear uncorrelated with mineral domains. Background non-Raman signals in SRS imaging are generally due to linear and/or non-linear absorption, and non-linear scattering. Background signals were seen in different thin sections as well as in the ground materials imbedded in resin. These seem to correlate, by simple visual inspection, with the degree of absorption. Many, but not all, of these responses appear to originate in volumes that are below the resolution limit of the optical microscope and often seem to be localized at the surface of the sample. Further research is required in order to identify the material origin of these absorptive signals. Methods for either avoiding (by using frequency or polarization modulation) or potentially taking advantage (i.e. for image contrast) of these background signal channels is a direction of our future research.

In summary, we have successfully compared SEM-EDS with SRS and SHG imaging in the comprehensive mapping of ankerite (Ca(Fe,Mg,Mn)(CO_3_)_2_) and barite (BaSO_4_) ores containing a central grain of quartz. Our study is an important step in the validation of multi-modal NLO imaging in mineralogy applications. We believe that this new approach to the study and rapid, preparation-free characterization of the multi-scale structural organization of natural ore and rock samples will be relevant to various aspects of mining and metallurgical processing.

## Materials and Methods

### Laser scanning microscopy

A schematic of the modulation-transfer optical microscopy set up is shown in Fig. 1(a). Further details of the apparatus are given in Houle *et al*.^20^. Briefly, a femtosecond laser system (InSight DS+, Spectra-Physics, USA) generates two synchronized pulse trains at 80 MHz. The first has a fixed central wavelength of 1040 nm and a transform-limited pulse duration of approximately 220 fs. The second is tunable over the range 680–1300 nm with a transform-limited pulse duration of approximately 100–120 fs. Both the pump and the Stokes pulses are linearly chirped (spread in time). The chirp is matched so that the instantaneous frequency difference remains the same across the two pulse envelopes. In this case, the femtosecond pulses are chirped with SF11 glass from a cross correlation width of ~300 fs to a cross correlation width of between 2–2.4 ps (full width half maximum). The final cross correlation width is measured at the microscope focus of the microscope by sum frequency generation using KDP (potassium dihydrogen phosphate) powder or non-degenerate two-photon fluorescence in fluorescein. A Pockels cell (350-160, Conoptics, USA), driven by a function generator (DS345, Stanford Research Systems, USA), produces a square wave amplitude modulation on the 1040 nm beam at a frequency of 1.4 MHz. The tunable arm traverses a variable delay, consisting of a retroreflector mounted on a linear stage. The two beams are combined on a dichroic mirror (1040dmbp, Chroma, USA) and then directed toward the laser scanning microscope.

The scanning module consists of a pair of galvanometer driven mirrors (Cambridge technology) and scan lens (50 mm Achromat–Thor Labs) which is mounted to the side of an IX71 inverted microscope (Olympus) and raster scans the focused laser for imaging. A dichroic beam splitter (720DCXXR, Chroma, USA), under the objective, passes the laser light and re-directs the visible signal, which is collected by the objective lens (20X 0.75 NA SPAPO, Olympus). The back-scattered second harmonic signal passes short pass (750SP, Chroma, USA) and band pass filters (520/10, Chroma) before its detection with a PMT module that has a built-in amplifier of approximately 200 kHz in bandwidth (Hamamatsu H10723-01).

Forward propagating light, after traversing the sample, is collected by a second objective lens (LUMPlanFI/IR, 40×, NA 0.8 water immersion, Olympus, Japan). The modulated beam is blocked while the unmodulated beam passes through a combination of a bandpass and notch filters (BrightLine 850/310, Semrock, USA and 1064-71 NF, Iridian, Canada). Typically, a few mW impinges onto a large area photodiode (FDS10X10, Thorlabs, USA), which is reverse biased at 50 V. The photo-current thus generated is amplified by a trans-impedance amplifier (DHPCA-100, Femto Messtechnik GmbH, Germany with a 10 MHz low pass filter enabled). The amplified signal is then fed into the lock-in amplifier (UHFLI, Zurich Instruments), which extracts the SRS signal at the reference frequency and phase provided by the function generator. A time constant of 20 μs is used. The relative phase between the signal and reference of the lock-in amplifier is adjusted to zero for the stimulated Raman loss (SRL) signal using a strong Raman signal from the symmetric stretch C-H bond mode around 2913 cm^−1^ of dimethyl sulfoxide (DMSO), Both the in-phase and the quadrature (90 degree out-of-phase) signals are accessed via the lock-in’s analog outputs. These signals are used as the inputs for the data acquisition in imaging.

For imaging, the average laser powers used at the sample are ranged approximately from 3 to 50 mW (corresponding to 0.038–0.625 nJ per pulse energy) in each laser arm. In most cases images of 256 × 256 pixels in size are acquired in 2.7 seconds (with the pixel dwell time of 38 microseconds). Co-ordination of the scanning with data acquisition is conducted through ScanImage software, which can also reconstruct the sample’s 3D (XYZ) position^52^. The images are processed using ImageJ/FIJI^53^.

### Scanning electron microscopy – energy-dispersive X-ray spectroscopy

Samples were examined on the Hitachi SU5000 analytical scanning electron microscope (SEM) equipped with an Oxford Instruments X-Max^N^ 80 mm energy dispersive X-ray (EDX) spectrometer. Backscattered electron (BSE) images and EDX analyses were carried out in low vacuum mode using an analysis chamber pressure of 50 Pa and accelerating voltage of 20 kV using AZtecEnergy EDX acquisition software. EDX spectra were collected over an energy range of 20 keV at a resolution of 10 eV per channel and a live time of 30 seconds per analysis. High resolution imaging was carried out in high vacuum mode using an accelerating voltage of 1 to 5 kV.

Large area mapping (LAM) was performed by obtaining a series of BSE images acquired at 1024 × 1024 with a dwell time of 10 µs per pixel. EDS X-ray maps were collected for each BSE image at 1024 × 1024 pixels while EDX spectra were acquired for each pixel at an energy range of 20 keV, 2048 channels, and 50 µs per pixel. Data from five images were averaged. LAM was set up using a perimeter overlap factor of 15% to ensure accurate reconstruction and stitching along individual image boundaries. The final images and associated X-ray maps were compiled into a single image to provide a large area composite of the areas of interest.

### Samples studied

Most samples used are standard polished 30 micron thin sections of carbonatite rare earth element ores^54^. The barite rock sample was from Ward’s Science (Rochester NY). For NLO microscopy, fragments of the barite rock were ground into random sub-mm-size particles by mortar and pestle and embedded in cyanoacrylate glue.
